# Rethinking mental healthcare: operationalising intersectionality through a community-driven social clinic model

**DOI:** 10.7189/jogh.15.03034

**Published:** 2025-09-19

**Authors:** Anuj Kapilashrami, George Kokkinidis, Marco Checchi, INNOVATE Consortium

**Affiliations:** 1 Centre for Global Health and Intersectional Equity Research, Institute of Public Health & Well-being, School of Health & Social Care, University of Essex, Colchester, UK; 2 Department of Management, Entrepreneurship and Digital Business, Cyprus University of Technology, Limassol, Cyprus; 3 Newcastle Business School, Northumbria University, Newcastle Upon Tyne, UK *Joint first authorship.

## Abstract

Here we examine the critical need for rethinking mental healthcare through a social clinic model, informed by intersectional equity perspective. Drawing on insights from the INtersectional Network Of community and stakeholder Voices, And research to Tackle (in)Equities (INNOVATE) project, we highlight significant gaps in current mental health services, including fragmentation, stigmatisation, and lack of person-centred care. Intersectionality recognises intersecting disadvantages that shape differential mental healthcare access and outcomes. By applying an intersectionality lens, we reveal how overlapping forms of disadvantage arising from social positions such as race and ethnicity, gender, geography, and immigration status interact with social drivers like socio-economic deprivation, legal status, and exclusion to create complex barriers to accessing care. For instance, young people in remote areas, asylum seekers with housing insecurity and experiencing disability, and those in socioeconomically deprived areas carry a disproportionate burden of poor mental health in Essex, UK. Social clinics, as autonomous, self-managed centres that combine medical care with social support and community participation, constitute a promising approach to reduce the gap. We argue for a community-led process of the integration of intersectionality into community engagement processes for establishing and implementing social clinics to build sustainable, inclusive, and responsive mental health services. We propose a five-step framework for operationalising this model, centred around co-design, collective action, and participatory governance, aiming to address power asymmetries and promote equity in mental health service delivery.

There is growing evidence that people-centred, community-driven primary healthcare (PHC) is ‛the most cost-effective, equitable and accessible route to extending health services to unreached populations’ [1]. PHC, distinct from primary level care, adopts a ‘whole-of-society’ approach with communities at its heart [2,3]. This approach brings centre stage a focus on empowerment of communities and their participation in decision-making related to their health. It promotes multisectoral action and integrated systems [4] that can deliver critical public health functions, necessary for tackling the social determinants of health [5].

Community-based care gained traction in mental health for its crucial role in promoting awareness, early identification, preventing disorders, reducing stigma, and supporting recovery [6]. Recognising the complex and interactive aetiology of mental health issues, which includes various socioeconomic, political and environmental factors, and the ‘treatment gap’, the World Health Organization recommends comprehensive, integrated mental healthcare. It also emphasises incorporating perspectives of service users and actively engaging communities. The global mental health gap refers to the gap between people who can access appropriate mental health and social care services and those who most need care and support for mental health conditions. Currently, nearly two-thirds of people affected by mental health conditions do not receive treatment.

Scholars advocate for shared decision-making, enhanced accessibility to services and a balanced care model that integrates hospital (clinical) and community-based care as cornerstones of such engagement [6,7]. Enhanced community involvement in service design, delivery and evaluation is core to these integrated care models.

Yet, studies examining community involvement in healthcare [7,8] reveal persistent evidence and implementation gaps. Further, even with good intentions, service planners and agencies frequently lack the resources, tools or knowledge to effectively involve communities in priority setting and service design for delivering comprehensive care. This gap is particularly more pronounced for low-income minority populations [9], resulting in calls to develop healthcare leadership that effectively engages communities to foster a strong commitment to high-quality mental healthcare [10].

This raises critical questions that we address in this viewpoint: What does a community-driven PHC model for mental health look like? How can such a model effectively reach populations experiencing multiple disadvantages? How can ground-up expertise from patients, affected communities, clinicians, and managers inform responsive and equitable service design?

To answer these questions, we first briefly summarise the key deficiencies in current mental healthcare provision in the UK and discuss the potential of community-led practices. We then reflect on the processes and practices of engaging communities, positing the idea of building ‘communities of care’ using a social clinics model. In the final section, we present an intersectionality-informed community engagement framework and outline practical steps and considerations in redesigning services around community needs.

## METHODS

We drew on a rapid review of literature on community engagement for (mental) healthcare and insights from the INtersectional Network Of community and stakeholder Voices, And research to Tackle (in)Equities INNOVATE project, which brings together diverse stakeholders and community voices in Essex, UK, to interrogate evidence gaps, co-design tools, and build capacities and improved solutions for reducing mental health disparities. We created various collaborative forums and utilised multiple participatory methods to connect and consult service planners, managers, and people with lived experience about gaps in evidence on burden, prevalence of poor mental health (especially depression and suicide) and access and utilisation barriers to services, with a particular focus on inequalities.

Specifically, we hosted ‘challenge laboratories’ to gauge stakeholder perspectives on key challenges in tackling risks and delivering mental healthcare for all and identify the population groups with the highest burden of mental ill health, the most common access barriers and their determinants (*e.g.* migration status, precarious employment and housing conditions, remote location and deprivation and poor digital literacy). Utilising arts-based methods of drawing and storytelling and asset mapping, we ran community workshops with five groups and engaged 30 people living with mental health issues. These workshops were effective in building trust with affected communities, helping them to open and share their life stories and experiences of accessing existing services and other community assets. In the final phase, through deliberative processes involving a legislative theatre and policy dialogue we disseminated key project findings and facilitated a lively policy discussion with key stakeholders from across the Essex care system. This phase was operative in eliciting a broad spectrum of responses and viewpoints on how to design more community and person-centred services.

## DEFICIENCIES IN MENTAL HEALTH SERVICES: COMMUNITIES AND STAKEHOLDERS’ PERSPECTIVE

Consistent with existing studies [11–13], our findings reveal major gaps in how mental health services are organised and delivered. Consultations and workshops with affected communities point to service fragmentation and a disconnect of services and programmes from marginalised groups as key barriers to addressing mental health inequalities.

At service level, professional expertise addressing complex issues like ageing, co-morbidities, and loneliness remains trapped within fragmented systems, while community-based solutions often remain localised and difficult to scale [14]. This is due to the fragmented approach to interventions that address the overlapping risk factors (*e.g.* poor transportation, housing, social isolation) in silos, and poor coordination and information sharing across systems. With respect to community initiatives in mental healthcare, a meta review [15] revealed a range of functions and improved outcomes, from raising awareness and equipping young people with life skills at population level to psychotherapy and educating families of persons living with acute mental health conditions. However, authors noted substantial variation in integrating community platforms into existing health systems and care structures, and limited user involvement in service design. Engagement was restricted largely to stakeholder consultations for cultural adaptations of pre-existing interventions.

These deficiencies were also noted by stakeholders and people with lived experiences engaged through INNOVATE. Participants reported growing distrust in mental health service providers and feelings of stigmatisation, attributing these to over-medicalisation, lack of person-centre care, labelling of people with mental health conditions and withdrawal of non-clinical support services.

I think I was let down by the system. I was in my 20s when I was diagnosed, which is quite frustrating. I've been to the doctors several times. They just put me on Prozac. They didn't really explain what it was just, you know, take this tablet, whatever it was. *– Lived experience participant, female.*

As part of the treatment I had, there was this therapy group, once every 2 weeks, where we discussed issues and that kind of thing, which I found helpful... I had been going for about 3 months and then told…in fact they did give us fair warning, about halfway through – ‘I’m sorry, this won’t be continuing in the new year, because of budget constraints. So therefore, we can’t accommodate you and these other people. *– Lived experience participant, male.*

My mother had to arrange private sessions (with counsellor), which were costly and beyond what she could afford. *– Lived experience participant, male.*

We also noted reports of peoples’ reluctance to seek help due to distrust and fear of potential adverse consequences from disclosing mental health conditions. During the challenge laboratory, two participants from the third sector who work closely with ethnic minority communities noted that fear and distrust are widespread and represent major barriers to accessing available services. They mentioned frequently being asked questions such as, ‘Will you take my children away?’ and ‘Will you tell social security?’. These accounts underscore the urgent need for collaborative initiatives that address these barriers by building trust and fostering a sense of community around mental health services.

Further, service improvements appeared to be hindered by austerity and a reduced spend on social support. In a pessimistic tone, indicative of their worry about the future state of mental health services in their areas, a participant said:

It’s a bleak future, I think. There was a community centre-up in Clacton. There was one just off, oh, the high street, there was a community centre behind that, but that’s gone. A friend of mine is up in Thurrock. That had a little community centre there, now gone. So, you know, things are being taken away. *– Lived experience participant in a community workshop, male.*

Current community interventions, including place-based and asset-based approaches, do not meet the diverse needs of individuals living in poverty, deprivation and facing multiple forms of marginalisation. A systematic review examining community participation in priority setting and health technology assessments found that, while various engagement methods exist, they frequently lack depth and continuity [8]. They also fail to understand or address prevalent inequalities, and the intersectional disadvantages experienced by those most at risk that impede their involvement and use of services. We examine these intersectional disadvantages in the following section.

Furthermore, power asymmetries – between planners and users, as well as within communities – are often overlooked [16], risking genuine community engagement and control [17], while shifting the responsibility for tackling social injustices onto the communities themselves. This depoliticised discourse tends to ignore the wider socioeconomic and political conditions that undermine well-being and widen inequalities [18–20].

## REDESIGNING SERVICES AROUND COMMUNITY NEEDS: INSIGHTS FROM USING AN INTERSECTIONALITY LENS

Understanding these inequalities and their determinants was a core premise and contribution of INNOVATE’s activities and was met by embedding the project in an intersectionality framework. Intersectionality moves beyond single-axis approaches that look at mental health inequalities in silos of age (young people) race/ethnicity, or gender (post-natal mental health of mothers). However, these intersections of social inequalities are insufficiently understood. A scoping review of intersectional inequalities in mental healthcare found that most studies focussed on gender, race/ethnicity, or socioeconomic status, and rarely on their intersectional effects [20].

We developed a novel ‛intersectional theory of change’ to guide its work, understanding that people’s (public) mental health is affected by a mix of multiple, interconnected identities, and social conditions. Its intersectional approach involved identifying most at-risk populations and specific underserved groups facing high mental health burdens and gaining a fuller understanding of key barriers to using mental health and support services. Here, we also conducted an intersectional analysis of data on depression and suicides, which we report in a forthcoming paper; examining how different ‛individual variables’ like race and ethnicity, gender, sexual orientation, geography, and socioeconomic status interact with ‛social drivers’ such as deprivation, (anti)immigration, austerity measures, political exclusion (especially in case of asylum seekers), and racism, making it harder for people to access mental health support, increasing access inequalities and isolation [21].

Participatory community workshops with people experiencing mental health challenges revealed that access barriers were most pronounced for individuals in deprived neighbourhoods, including coastal communities, young people – especially sexual and gender diverse populations and those in remote areas, and asylum seekers with disabilities. For instance, for people living in coastal towns, the intersectional approach showed how geographic isolation (*i.e.* spatial factors) interacts with economic deprivation and limited access to services, impacting their mental well-being. We found that residents in these areas often feel ‛excluded’ and ‛not listened to’ by service planners.

In the case of children and young people, INNOVATE’s intersectional lens revealed how age intersects with factors like social media use or difficult circumstances in their families to create complex mental health issues. The project noted a rapid increase in mental health problems among children and young people in areas like Basildon, Southend, and Tendering, highlighting the need for age-specific and early years interventions that also consider other intersecting factors.

For refugees and asylum seekers, as well as people experiencing homelessness, their legal status and housing insecurity interact with prior experiences of trauma to create multifaceted mental health challenges. The project found that these groups often face compounded barriers to accessing mental health support, including a lack of eligibility for public funds, language barriers, and high distrust combined with low awareness of public services, considering the growing anti-immigrant sentiment. Access barriers were particularly pronounced for new migrants from minoritised ethnic groups and asylum seekers. Many reported a significant lack of awareness concerning available community assets and well-being resources, especially green and blue spaces, along with the various structural barriers to accessing these [22], and weak social networks and ties [23].

In community workshops held with a group of asylum seekers temporarily residing in a hostel, two young participants living with disability, separate from their families, and suffering from depression shared:

I was very happy when I first came to the UK. Then I had an accident – a car hit me and ran. I was in coma for one month, hospitalised for three months. After that, I don’t feel well anymore. I don’t go out. I don’t like to sit with anyone. I don’t like my life anymore. *– Lived experience participant, male, asylum seeker with disability.*

I want to go to the beach, but I cannot walk long. When I walk halfway, I need to stop to rest. I’m disabled. I can’t afford a taxi either. *– Lived experience participant, male, asylum seeking with disability.*

Other participants reported a lack of services tailored to their specific demographic, suggesting targeted services generate critical gaps.

if you’re a male between the ages of 25 and 55, there’s nothing out there for you, there’s absolutely nothing out there. I mean, I have looked on the Internet, I have [gone] up to Brentwood, looked in the Library, and all sorts of things, trying to get groups that I could affiliate with, that effectively would get me out of here. And there’s nothing, there’s absolutely nothing.* – Lived experience participant in community workshops, male.*

In a nutshell, the barriers expressed by affected communities included accessibility issues such as distance to health facilities, lack of time and money, unawareness of available services, and feelings of loneliness and marginalisation.

To address these challenges and provide more inclusive and equitable mental health services tailored to community needs, a strong consensus emerged on the necessity of an intersectionality lens. By better understanding and addressing the multiple and intersecting social disadvantage that is driving people’s mental health experience, intersectionality can help create healthcare services that are both inclusive and responsive to the mental health needs of those who are most severely and multiply deprived [24].

Interest in such inclusive and sustainable ways to address mental health issues is growing even at institutional and policy levels. This is evident in initiatives such as the integrated neighbourhood teams in the NHS, but also emerged prominently in the policy dialogue in INNOVATE. However, participants reported two critical impediments to such inclusive responsive service design: poor stakeholder collaboration and a lack of appropriate and diverse data to assess which populations are most at risk and for design of services to reach them effectively. An indicative example, as reported by one of our participants in the challenge laboratory, is the new National Health Service five-year plan for the local integrated care boards that had not ‛a single reference to refugees and asylum seeking’. They stressed the need to create initiatives that will put these communities at the heart of all their activities.

To this end, Béhague and colleagues’ [25] study on marginalised youth in Brazil offers a compelling argument for rethinking mental health services in terms of community empowerment and political engagement. They illustrate how therapy can be a tool for collective identity-building, critique and resistance when grounded in psychodynamic and narrative approaches as opposed to the traditional behavioural framing of mental health treatments. Central to these alternative approaches is the enhancement of community engagement and service delivery through services that recognise the role of institutions and power dynamics, invest in justice-oriented approaches to care, avoid pathologising behaviour and ultimately act as incubators for collective agency. How this vision can be achieved remains a core dilemma for policy researchers, planners, and implementers.

## BUILDING COMMUNITIES OF CARE: THE PROMISE OF SOCIAL CLINICS

In recent years, a wave of grassroots initiatives guided by the principles of user-control and user-benefit have gained traction, attempting to reorient community engagement and participation to its former collective and political dimension. To this end, social clinics constitute a prominent model of community-led organising of healthcare [22,26].

Social clinics are autonomous and self-managed organisations that act as one-stop centres bring together medical professionals, patients, and local communities to co-design services and address social determinants of health. They combine General Practice services, psychiatric care, welfare support, and counselling, while actively promoting community development from an equity lens.

The effectiveness of social clinics as vehicles for developing communities of care is strongly evident in Da Mosto and colleagues’ [27] study across three social clinics located in France, Italy, and Greece. Building caring systems is not a one-off activity and requires genuine collective efforts. They offer a detailed account of the community engagement processes centred around the intersections of micro-level (medical practices and patient-professional interactions), meso-level (organisational design and co-creation processes) and macro-level (alignment with broader movements and political economic struggles) practices of engagement. At a micro-level, we have the example of the Social Clinic of Solidarity (KIA) in Greece, a social clinic whose reframing of the doctor-patient role emphasises equality and shared responsibility. They have introduced a range of initiatives such as cooperative dentistry, integrative medicine team to disrupt conventional hierarchies and build a sense of community through egalitarian, experimental engagement in healthcare practices. At a meso-level, *Village 2 Santé* in France identified participation as an ongoing process embedded in the clinic’s structure, tailoring services based on community needs assessment and introducing informal spaces for interaction and relationship-building. This approach nurtured dynamic and inclusive service delivery. Finally, at a macro level, the *Microclinica Fatih* clinic in Italy acts as a hub for activism, expanding healthcare beyond treatment into structural critique and political resistance. Drawing on these examples, we observe that community engagement in social clinics is not just about enhancing service delivery, but also radically reimagining power, identity, and justice in health. Each case illustrates a distinct yet interconnected path towards building ‘communities of care’ through initiatives that are bottom-up, participatory, and deeply political. Checchi and Kokkinidis [28] further reflect on the processes required to create ‘communities of care’ that offer a transformative space where power is reconfigured, participation is embodied, and care becomes a political act.

Social clinics are not without their challenges. Some of them are generic, such as those of resource constraints and how to sustain the clinical component (*e.g.* staffing), while others are more context-specific, such as institutional recognition and integration with the public health system. These issues were also evident in the above cases [26]. However, we note that these initiatives are trying to address some of these problems. For example, they created solidarity networks and sought alternative sources of funding (*e.g.* fundraising, donations, community events) in response to resource constraints and sustainability.

Furthermore, the social clinics’ character, practices, and goals are influenced by their origin, acceptability, and support from the state, and their integration into the public health system. They may also be initiated by either social movements, third sector organisations, or other institutional actors, which in turn impacts their objectives and practices. Those deriving directly from social movements tend to be more flexible in their approach, rooted in the communities they serve and put more emphasis on solidarity and health as a political matter, while those led by formal institutions tend to be more top-down, with bureaucratic structures and perceive their role in a more philanthropic manner. Within this context, social clinics that emerge from social movements are presented with a challenge that evolves around questions of integration and autonomy. This challenge is addressed by putting in place organisational processes and practices that will safeguard the autonomous and community-led character of social clinics while collaborating with the public health system. The case of *Village 2 Santé* described above is a case in point, where system integration has been achieved without compromising the principles of horizontality, inclusive decision-making processes and community-led service provisions [27]. Having community empowerment at their core, social clinics, like *Village 2 Santé*, enhance access to community assets and ensure services are tailored to local needs through bottom-up approaches, including the co-creation of services and the adoption of participatory healthcare practices.

## OPERATIONALISING SOCIAL CLINICS THROUGH AN INTERSECTIONALITY-INFORMED COMMUNITY ENGAGEMENT PROCESS

While involvement of communities, especially those left behind in priority setting and design, is a core premise of social clinics, there is little guidance on how to do so, and how this process can incorporate an intersectionality perspective [29]. We offer a framework emphasising progressive engagement and participation of communities in designing and implementing health services and interventions. The framework, initially developed by Kapilashrami (unpublished data material) for the WHO Eastern Mediterranean Regional Office was subsequently adapted for INNOVATE through consultations with partners and stakeholders in Essex. It was informed by a systematic review of evidence on community participation, highlighting the key barriers to meaningful participation typologised as institutional, procedural, technical, and structural barriers [30].

The framework delineates depth, frequency, and stages of engagement. The stage of engagement is depicted as moving progressively from simply ‘informing’ the community to ‘empowering’ them, across all stages of service design: data, dialogue, decision, review, evaluation, advocacy and regulation. Ensuring sustainability requires establishing institutional mechanisms (such as community reference groups or a community safety partnership (CSP) committee) that would enhance the frequency of community engagement over time.

**Figure 1:**
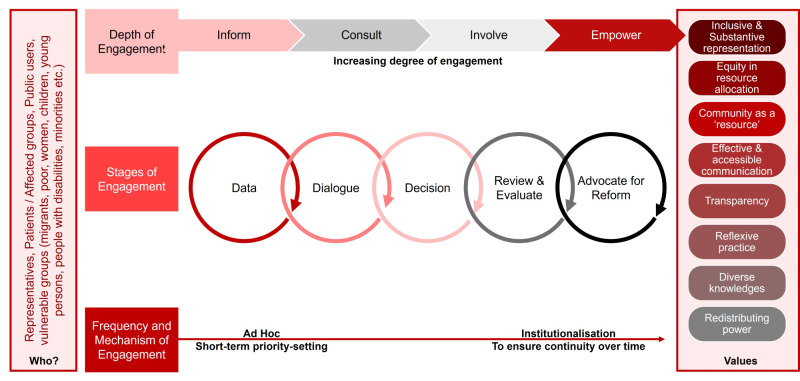
Framework for progressively enhancing community participation in priority setting and service design (from [31], p. 40).

Depth, stages, and frequency of engagement are interlinked (**Figure 1**). Our research revealed a strong demand for integrating local knowledge in designing community-based mental healthcare and fostering collaboration at every stage, from planning and co-design to service implementation and evaluation [23]. It stressed the importance of ongoing collaboration in moving from basic levels of engagement (to ‘inform’) through to full empowerment, and identifies key principles to guide such collaboration for long-term impact and sustainability. As reiterated by participants during consultations on the framework, these included: trusting and valuing community as a resource, respecting diverse knowledges including experiential insights, ensuring equity in representation and outcomes, and embracing reflexive practice.

Furthermore, sustained engagement can be enabled through social clinics as they provide primary care while also operating as community hubs, embedded within the communities they serve. Key considerations to design a sustainable social clinics model include:

1. A cooperative model of organising that emphasises the use of creative and participatory methods, inclusive language, information sharing and the reorientation of ‘expert’ knowledge in a more horizontal and symmetrical way.
2. Engagement with the local communities not as a one-off exercise but as a continuous process of collaboration, across its various stages of ‘data’, ‘dialogue’, ‘design’, ‘evaluation’, ‘advocacy’, and reform.
3. Development of new partnerships to encourage greater cooperation across previously fragmented services and a shared knowledge hub that synthesises evidence to inform priorities.
4. Linking services with community assets and activities to build social connectedness and resilience.
5. Embedding the above processes and organising practices in the key principles of intersectionality – that requires openness, receptiveness, trust and respect for all knowledge systems alongside addressing power dynamics.

Incorporating these principles, we propose a five-step operational framework to establish sustainable partnerships among key stakeholders. These steps, which will be detailed in a forthcoming paper along with guiding questions, are designed to foster effective collaboration and long-term improvements in mental healthcare.

1. Conduct a situational analysis to help identify the most appropriate type of social clinic for the specific context, map community assets, services, needs, and existing mechanisms for engaging communities.
2. Co-design a community engagement plan that would recognise the complexities, social inequalities, and power structures within these communities. This requires careful design of participatory organisational practices and inclusive decision-making processes that promote collaboration.
3. Establish a community-led model of inclusive care by encouraging collective action, and user participation across all activities of the social clinics. This involves creating flexible structures and processes to minimise bureaucratic barriers and exclusion, while ensuring that teams reflect the diversity and experiences of the community.
4. Build a sustainable system for community engagement by embedding actions in participatory structures (*e.g.* use of general assemblies). Additionally, support network infrastructures and multi-stakeholder collaborations including partnerships between medical associations, social movements and city councils.
5. Develop processes for ongoing evaluation of participation, equity of access/ reach, and outcomes to support sustainable reforms and improvements.

We argue that an intersectionality-informed framework – utilising social clinics’ participatory governance, flexible organisational structures and community empowerment – offers a promising model for equitable and sustainable health interventions grounded in solidarity, participation, and justice-oriented approaches to care.

## CONCLUSIONS

We propose a reimagining of mental healthcare through a social clinics model of organising that applies intersectionality to design inclusive, participatory and integrated services [21,31–33].

A core contribution of INNOVATE has been advancing the application of intersectionality theory to critically revisit mental health research and interventions. Our exploration of an intersectional approach to mental health emphasises the importance of:

1. A holistic understanding that moves beyond isolated treatment of single health issue to examining the syndemic co-existence of multiple conditions within the context of socioeconomic and political systems that underpin these.
2. Identifying and tackling the complex interactions between social determinants – including diverse social identities and environments – and the mental health risks they influence (*e.g.* substance use, childhood adversity, displacement and conflict-related trauma).

Intersectionality can help reveal the pathways through which mental health inequalities are formed [34], ranging from individual-level factors of adverse childhood experiences, developmental challenges, and risk exposures to broader social and structural conditions like racism, ableism, and xenophobia that manifest into a lack of social support and pervasive stigma and discrimination. The social clinic model, on the other hand, can empower service planners and providers to better address the systemic barriers to accessing care that place those most disadvantaged at greatest risk of poor mental health. In turn, policymakers can benefit from gaining more nuanced context-specific insights into these inequalities that guide resource allocation and action.

Combining the equity promise of intersectionality and the transformative power of social clinics to act on social determinants through partnerships can help deliver the ‘whole of society’ vision and define the future of healthcare systems for healthier societies.

## References

[1] Allen LN Pettigrew LM Exley J Nugent R Balabanova D Villar-Uribe M The role of primary health care, primary care and hospitals in advancing universal health coverage. BMJ Glob Health. 2023;8:e014442. 10.1136/bmjgh-2023-014442 38084496 PMC10711840

[2] Hanson K Brikci N Erlangga D Alebachew A De Allegri M Balabanova D The Lancet Global Health Commission on financing primary health care: putting people at the centre. Lancet Glob Health. 2022;10:e715–72. 10.1016/S2214-109X(22)00005-5 35390342 PMC9005653

[3] Palmer VJ The participatory zeitgeist in healthcare: it is time for a science of participation. J Particip Med. 2020;12:e15101. 10.2196/15101 33064092 PMC7434075

[4] Starfield B Shi L Macinko J Contribution of primary care to health systems and health. Milbank Q. 2005;83:457–502. 10.1111/j.1468-0009.2005.00409.x 16202000 PMC2690145

[5] Hiam L Klaber B Sowemimo A Marmot M NHS and the whole of society must act on social determinants of health for a healthier future. BMJ. 2024;385:e079389. 10.1136/bmj-2024-079389 38604669

[6] Thornicroft G Mehta N Clement S Evans-Lacko S Doherty M Rose D Evidence for effective interventions to reduce mental-health-related stigma and discrimination. Lancet. 2016;387:1123–32. 10.1016/S0140-6736(15)00298-6 26410341

[7] Alwan A Yamey G Soucat A Essential packages of health services in low-income and lower-middle income countries: what have we learnt? BMJ Glob Health. 2023;8:e010724. 10.1136/bmjgh-2022-010724 36657807 PMC9853117

[8] Arthur M Saha R Kapilashrami A Community participation and stakeholder engagement in determining health service coverage: a systematic review and framework synthesis to assess effectiveness. J Glob Health. 2023;13:04034. 10.7189/jogh.13.04034 37166063 PMC10173679

[9] Mendel P O’Hora J Zhang L Stockdale S Dixon EL Gilmore J Engaging community networks to improve depression services: A cluster-randomized trial of a community engagement and planning intervention. Community Ment Health J. 2021;57:457–69. 10.1007/s10597-020-00632-5 32430557 PMC7906961

[10] Patel KK Butler B Wells KB What is necessary to transform the quality of mental health care. Health Aff (Millwood). 2006;25:681–93. 10.1377/hlthaff.25.3.681 16684732

[11] Ådnanes M Steihaug S “You never know what happens next” - Young adult service users’ experience with mental health care and treatment through one year. Int J Integr Care. 2016;16:5. 10.5334/ijic.2435 28435418 PMC5350637

[12] Bjørkquist C Hansen GV Coordination of services for dual diagnosis clients in the interface between specialist and community care. J Multidiscip Healthc. 2018;11:233–43. 10.2147/JMDH.S157769 29805264 PMC5960238

[13] Trane K Aasbrenn K Rønningen M Odden S Lexén A Landheim A Integration of care in complex and fragmented service systems: experiences of staff in Flexible Assertive Community Treatment teams. Int J Integr Care. 2022;22:17. 10.5334/ijic.6011 35651735 PMC9139156

[14] Fakoya OA McCorry NK Donnelly M Interventions Aimed at Alleviating Loneliness and Social Isolation among the Older Population: Perspectives of Service Providers. Health Soc Care Community. 2023;2023:1–8. 10.1155/2023/5613153

[15] Kohrt BA Asher L Bhardwaj A Fazel M Jordans MJD Mutamba BB The Role of Communities in Mental Health Care in Low- and Middle-Income Countries: A Meta-Review of Components and Competencies. Int J Environ Res Public Health. 2018;15:1279. 10.3390/ijerph15061279 29914185 PMC6025474

[16] Esposto E, Moini G. Participatory governance and healthcare: opportunities and perils. In: Battisti A, Marceca M, Iorio S, editors. Urban Health. Cham, Germany: Springer; 2020. p. 9–18.

[17] Wallerstein N Rosilda M Minkler M Akerman M Reclaiming the social in community movements: perspectives from the USA and Brazil/South America: 25 years after Ottawa. Health Promot Int. 2011;26:ii226. 10.1093/heapro/dar077 22080077 PMC6287427

[18] Popay J Whitehead M Ponsford R Egan M Mead R Power, control, communities and health inequalities I: theories, concepts and analytical frameworks. Health Promot Int. 2021;36:1253–63. 10.1093/heapro/daaa133 33382890 PMC8515177

[19] Fagrell Trygg N Gustafsson PE Månsdotter A Languishing in the crossroad? A scoping review of intersectional inequalities in mental health. Int J Equity Health. 2019;18:115. 10.1186/s12939-019-1012-4 31340832 PMC6657170

[20] Aziz R Kapilashrami A Majdzadeh R Exploring the inequalities experienced by health and care workforce and their bases – a scoping review protocol. PLoS One. 2024;19:e0302175. 10.1371/journal.pone.0302175 38625874 PMC11020832

[21] Kapilashrami A Marsden S Examining intersectional inequalities in access to health (enabling) resources in disadvantaged communities in Scotland: advancing the participatory paradigm. Int J Equity Health. 2018;17:83. 10.1186/s12939-018-0797-x 30244682 PMC6151920

[22] Cabot H ‘Contagious’ solidarity: Reconfiguring care and citizenship in Greece’s social clinics. Soc Anthropol. 2016;24:152–66. 10.1111/1469-8676.12297

[23] Kapilashrami A, Kokkinidis G, Bennet E, Haworth S. Innovate Challenge Lab Report. Essex, UK: University of Essex; 2022. Available: https://innovate4mh.org/wp-content/uploads/2023/11/Challenge-Lab-Report.pdf. Accessed: 23 April 2025.

[24] Kapilashrami A Hankivsky O Intersectionality and why it matters to global health. Lancet. 2018;391:2589–91. 10.1016/S0140-6736(18)31431-4 30070211

[25] Béhague DP Gonçalves H da Cruz SH de Cruz L Horta BL Lima NP The politicizing clinic: insights on ‘the social’ for mental health policy and practice. Soc Psychiatry Psychiatr Epidemiol. 2024;59:523–36. 10.1007/s00127-023-02573-2 38108834 PMC10944422

[26] Teloni D Adam S Solidarity Clinics and social work in the era of crisis in Greece. Int Soc Work. 2018;61:794–808. 10.1177/0020872816660604

[27] Da Mosto D Vallerani S Kokkinidis G Checchi M Giaimo S Adami E Building communities of health: the experience of European social clinics. Community Dev J. 2023;58:595–613. 10.1093/cdj/bsad015

[28] Checchi M, Kokkinidis G. From stakeholders to communities of care. In: Weik E, Land C, Hartz R, editors. The Handbook of Organizing Economic, Ecological and Societal Transformation. Boston, Massachusetts, USA: De Gruyter; 2024. p. 257–74.

[29] Fagrell Trygg N Månsdotter A Gustafsson PE Intersectional inequalities in mental health across multiple dimensions of inequality in the Swedish adult population. Soc Sci Med. 2021;283:114184. 10.1016/j.socscimed.2021.114184 34229136

[30] Arthur M Saha R Kapilashrami A Systematic review of community participation and stakeholder engagement in priority setting for health services: assessing effectiveness and equity. J Glob Health. 2023;13:04034. 10.7189/jogh.13.04034 37166063 PMC10173679

[31] Kapilashrami A, Quinn N, Das A. Advancing Health Rights and Tackling Inequalities: Interrogating Community Development and Participatory Praxis. Bristol, UK: Policy Press; 2025.

[32] Kokkinidis G Checchi M Power matters: posthuman entanglements in a social solidarity clinic. Organization. 2023;30:288–306. 10.1177/1350508420973304

[33] Chadd K Malik M Kapilashrami A Operationalising participatory action research to evaluate early years’ population health services. BMC Public Health. 2025;25:947. 10.1186/s12889-025-22183-8 40065342 PMC11895270

[34] Funer F Admitting the heterogeneity of social inequalities: intersectionality as a (self-) critical framework and tool within mental health care. Philos Ethics Humanit Med. 2023;18:21. 10.1186/s13010-023-00144-6 38001488 PMC10668443

